# Ferroelectric Switching of Vinylidene and Trifluoroethylene Copolymer Thin Films on Au Electrodes Modified with Self-Assembled Monolayers

**DOI:** 10.3390/ma7096367

**Published:** 2014-09-08

**Authors:** Naoto Tsutsumi, Tomotaka Kitano, Kenji Kinashi, Wataru Sakai

**Affiliations:** Macromolecular Science and Engineering, Kyoto Institute of Technology, Matsugasaki, Sakyo, Kyoto 606-8585, Japan; E-Mails: tomojin3933@gmail.com (T.K.); kinashi@kit.ac.jp (K.K.); wsakai@kit.ac.jp (W.S.)

**Keywords:** ferroelectric switching, self-assembled monolayer, remanent polarization, coercive field, switching speed, vinylidene fluoride and trifluoroethylene copolymer

## Abstract

The ferroelectric switching characteristics of a vinylidene fluoride and trifluoroethylene copolymer were significantly changed via the chemical modification of a gold electrode with an alkanethiol self-assembled monolayer (SAM). The alkanethiol SAM-Au electrode successfully suppressed the leakage current (dark current) from the electrode to the bulk ferroelectric. Smaller leakage currents led to the formation of an effective electric field in the bulk ferroelectric. At switching cycles ranging from 10 to 100 kHz, significant changes in the ferroelectric properties were observed. At 100 kHz, a remanent polarization (Pr) of 68 mC·m^−2^ was measured, whereas a very small Pr value of 2.4 mC·m^−2^ was measured for the sample without a SAM. The switching speed of the SAM-Au electrode is as twice as fast as that of the unmodified electrode. A large potential barrier was formed via the insertion of a SAM between the Au electrode and the ferroelectric, effectively changing the ferroelectric switching characteristics.

## 1. Introduction

Ferroelectric polymers such as poly(vinylidene fluoride) (PVDF) and its copolymer with trifluoroethylene (P(VDF/TrFE)) have been extensively investigated because of the spontaneous polarization due to the β-phase crystallite and their ferroelectric switching. Ferroelectricity is the property of spontaneous polarization reversal in the presence of an alternating electric field (ferroelectric polarization switching). Organic ferroelectric switching of P(VDF-TrFE) has attracted attention in the field of nonvolatile memory devices [1,2,3,4]. Khan *et al.* [2] have demonstrated non-volatile organic ferroelectric memory on banknotes and high-performance organic conductive PEDOT-PSS (Poly(3,4-ethylenedioxythiophene) Polystyrene sulfonate ) electrodes.

We previously measured ferroelectric switching at frequencies between 10 and 100 kHz and reported the ferroelectric properties of P(VDF-TrFE) copolymer thin films [5]. To obtain sufficient remanent polarization at a higher cycling frequency of 100 kHz, we applied a high electric field of 278 MV·m^−1^ to the P(VDF-TrFE) copolymer [5]. However, this field is too high to apply in electronic devices. To lower the requisite electric field, two major approaches can be applied: a reduction in the interaction between the ferroelectric crystals, *i.e.*, controlling the packing density of crystals and the domain size, or modification of the electrodes to change their ferroelectric switching performance. Chen *et al.* [4] investigated the effect of reduced ferroelectric domains on switching performance in a terpolymer of VDF, TrFE and chlorofluoroethylene (CFE).

In this study, several types of self-assembled monolayers were introduced between a gold electrode and the ferroelectric to change the ferroelectric switching properties of P(VDF-TrFE). For comparison, a PEDOT-PEG (Poly(3,4-ethylenedioxythiophene), bis-poly(ethyleneglycol)) lauryl organic conductive layer was inserted between the gold electrode and the ferroelectric bulk. We evaluated the ferroelectric switching performances of the coercive field, the remanent polarization, and the switching speed at different amplitudes of the applied electric field and at different alternating electric field frequencies of 10–100 kHz.

## 2. Experimental Section

### 2.1. Materials

A copolymer of vinylidene fluoride 60 mol% and trifluoroethylene 40 mol% (P(VDF/TrFE) (60/40)) was used as a ferroelectric polymer. Two types of interlayer were introduced between the electrode and the polymer film: one was an organic conductive layer of PEDOT-PEG lauryl, which was purchased from Sigma-Aldrich, St. Louis, MO, USA, and the other was an alkanethiol self-assembled monolayer (alkanethiol SAM). 1-Butanethiol, 1-decanethiol, 1-dodecanethiol, and 1-octadecanethiol were used as alkanethiol SAMs. P(VDF-TrFE) was dissolved in methyl-ether-ketone (MEK) to prepare the polymer solution for spin coating.

### 2.2. Sample Preparation

The normal cell type was a sandwiched cell. P(VDF-TrFE) was dissolved in MEK to prepare a 4.5-wt% P(VDF-TrFE) MEK solution. The P(VDF-TrFE) MEK solution was spin-coated at 1000 rpm for 30 s onto a 5 mm ϕ gold electrode evaporated onto a silicon substrate; the resulting sample was subsequently thermally annealed at 140 °C for 2 h under a vacuum. Another tiny gold electrode was evaporated onto an annealed P(VDF-TrFE) thin film using a 117 μm × 117 μm mesh mask (Au sample).

PEDOT-PEG lauryl was dissolved in nitromethane to prepare a 0.7-wt% PEDOT-PEG lauryl nitromethane solution. The PEDOT-PEG lauryl nitromethane solution was spin-coated at 1000 rpm for 30 s onto a 5 mm ϕ gold electrode evaporated onto a silicon substrate; the P(VDF-TrFE) MEK solution was subsequently spin-coated at 1000 rpm for 30 s. The resulting sample was thermally annealed at 140 °C for 2 h under a vacuum. The PEDOT-PEG lauryl nitromethane solution was spin-coated onto the P(VDF-TrFE) thin film at 1000 rpm for 30 s, and a tiny gold electrode was evaporated onto the sample using a 117 μm × 117 μm mesh mask (PEDOT-PEG sample). 

The alkanethiols (1 × 10^−3^ M) were dissolved separately in ethanol. A 5 mm ϕ gold electrode evaporated onto a silicon substrate was soaked in an alkanethiol ethanol solution for one day. After the electrode was rinsed with ethanol, the SAM layer was dried under ambient conditions. The P(VDF-TrFE) MEK solution was then spin-coated onto the SAM-coated Au substrate at 1000 rpm for 30 s. Next, the sample was thermally annealed at 140 °C for 2 h. Finally, another gold electrode was evaporated onto the annealed P(VDF-TrFE) thin film using a 117 μm × 117 μm mesh mask (SAM sample). We labeled the samples prepared using 1-butanthiol, 1-decanethiol, 1-dodecanethiol, and 1-octadecanethiol as SAM4, SAM10, SAM12, and SAM18, respectively. The resulting area of the bottom gold electrode was 5 mm ϕ, and the area of the top gold electrode was 117 × 117 μm^2^. The thickness of the gold electrodes was 40 nm. Before the gold top electrode was deposited, the P(VDF-TrFE) film was thermally annealed at 140 °C for 2 h to induce crystallization.

### 2.3. Characterization

The contact angles of the SAM films were measured. The surface topologies, roughnesses and film thicknesses were measured using a Nano-R atomic force microscope (Pacific Nanotechnology, Santa Clara, CA, USA). The surface topographies were measured using contact mode. The root-mean-square average surface roughness (*R*_RMS_) was evaluated using the Nano-R software (Pacific Nanotechnology, Santa Clara, CA, USA).

### 2.4. Ferroelectric Measurements

The ferroelectric switching was measured using a FCE-1A ferroelectric measurement system (Toyo Corporation, Tokyo, Japan) in combination with an atomic force microscope. A conductive probe chip was used in contact mode to measure the switching current in the sample film. The applied voltage was in the range of 2.5–30 V, with a step of 0.1 V.

## 3. Results and Discussion

### 3.1. Contact Angle

To confirm the fabrication of the SAM layers on the gold electrodes, the contact angle of water was measured on a gold electrode and on the SAM-coated gold electrodes. The contact angles are listed in Table 1. The contact angle of water on the gold electrode was 76.8°, whereas those on the SAM layers were 96.5°, 113.2°, 111.6° and 106° for SAM4, SAM10, SAM12, and SAM18, respectively. These results imply that hydrophobic SAM layers were fabricated on the gold electrodes.

### 3.2. Atomic Force Microscope (AFM) Measurements

**Figure 1:**
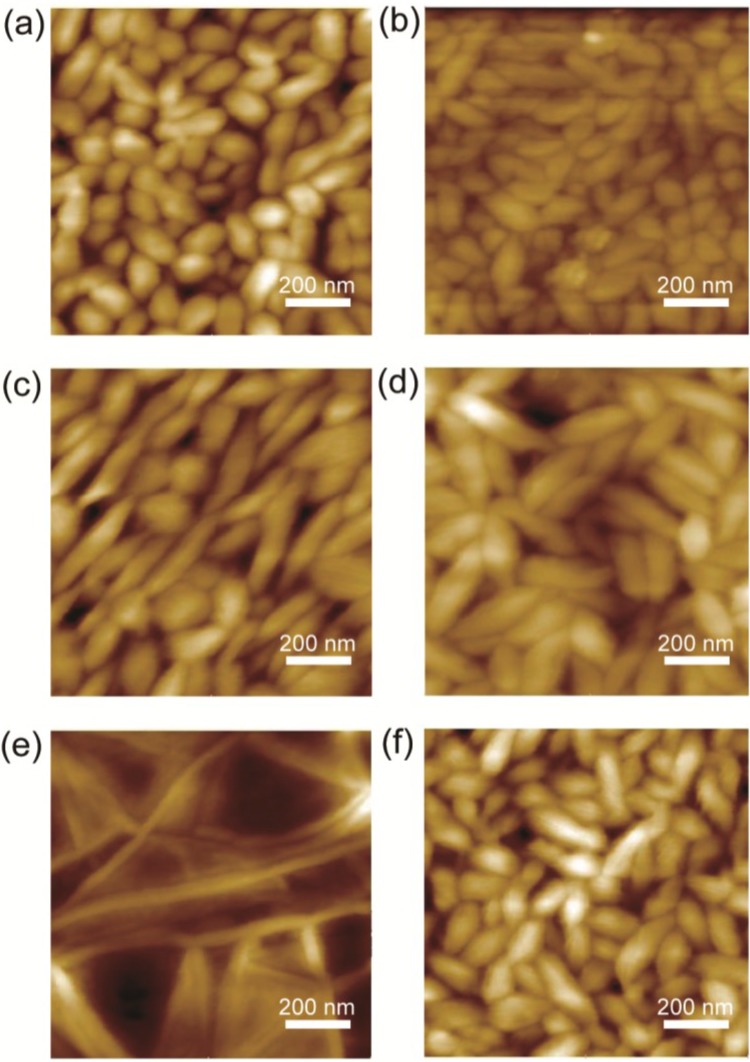
AFM images (1 μm × 1 μm) of the surface morphology of P(VDF-TrFE) on (**a**) a bare Au electrode and on Au electrodes coated with (**b**) PEDOT-PEG lauryl; (**c**) SAM4; (**d**) SAM10; (**e**) SAM12 and (**f**) SAM18.

The film thicknesses were measured using an AFM; the results are listed in Table 1. The film thicknesses of the P(VDF-TrFE) were 420, 470, 300, 357, 355, and 317 nm for the samples with Au only, PEDOT-PEG lauryl, SAM4, SAM10, SAM12, and SAM18, respectively. Figure 1 shows the AFM topography for the respective samples. The samples, with the exception of SAM12, exhibit grain-like crystallites that are uniformly developed; the SAM12 sample exhibits needle-like crystallites. The authors of previous studies [5,6] have also reported grain-like crystallites in ferroelectric P(VDF-TrFE) thin films.

The surface roughnesses were evaluated because the roughness sometimes directly affects the uniformity of the applied electric field inside the sample. A flat, uniform surface is ideal. The *R*_RMS_ values of the samples are summarized in Table 1. In the case of the SAM4 sample, a low *R*_RMS_ that was almost the same as that of the unmodified Au electrode was measured. An increase in the number of methylene groups in the SAM led to a rougher surface; the electrode coated with PEDOT-PEG exhibited the roughest surface.

**Table 1:** Contact angles, thicknesses and roughnesses (*R*_RMS_) for each sample.

Sample	Contact angle (°)	Thickness (nm)	*R*_RMS_ (nm)
Au	76.8	420	1.45
PEDOT-PEG lauryl	-	370	13.0
SAM4	96.5	300	1.47
SAM10	113.2	357	2.98
SAM12	111.6	355	2.63
SAM18	106	317	6.31

### 3.3. Ferroelectric Switching

Similar to the method discussed in our previous report [5], we evaluated the switching characteristics of the organic ferroelectrics by switching the current applied to the tiny Au electrode using a sinusoidal voltage and an AFM probe. The frequency of the applied voltage ranged from 10 to 100 kHz.

(1) $$J(t)=\frac{\text{d}P}{\text{d}t}+{\text{εε}}_{0}\frac{\text{d}E}{\text{d}t}+\frac{E}{\text{ρ}}$$

where *P* is the polarization, ε is the dielectric constant, ε_0_ is the permeability in vacuum, *E* is the electric field, and ρ is the resistivity. This equation is simplistic because the higher-order term for polarization and the nonlinear term for resistivity are not included. Therefore, this equation cannot be used to explain these phenomena, including the higher-order terms. However, in the present case, these higher-order terms are negligibly small, so an analysis using Equation (1) is adequate.

The switching current, *J*(*t*), is the summation of the polarization current term, the capacitance term, and the resistance term. After subtracting the capacitance and resistivity terms from the total current *J*(*t*), we evaluated the polarization. The hysteresis curve of the polarization was obtained via the integration of d*P/*d*t* with time. The switching characteristics were evaluated on the basis of the coercive field (Ec) and the remanent polarization (Pr).

**Figure 2:**
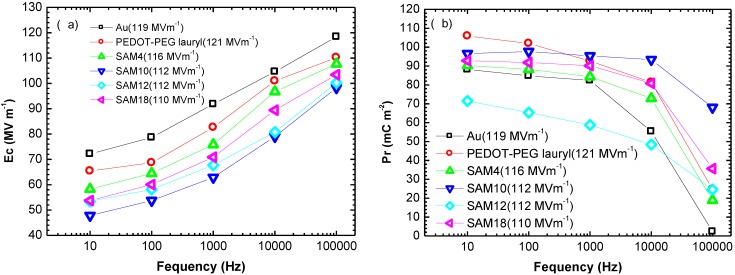
Plots of (**a**) Ec and (**b**) Pr as functions of the frequency of the alternating electric field for each sample.

The Ec and Pr values for each sample are plotted as functions of the switching frequency in Figure 2. All Ec and Pr values were measured in a saturated electric field of 110–120 MV·m^−1^. The results in Figure 2 show that the Ec and Pr values were significantly affected by the interlayer between the gold electrode and by the P(VDF-TrFE) thin film. The interlayer between the gold electrode and the P(VDF-TrFE) thin film changed the switching characteristics. Notably, the SAM10 sample exhibited a lower Ec of 48 MV·m^−1^, whereas the Au sample exhibited a higher Ec of 72 MV·m^−1^ during cycling at 10 Hz. During cycling at 100 kHz, the Ec values were not affected by the interlayer, but the Pr value was significantly affected by the interlayer of PEDOT-PEG lauryl and by the SAMs. This effect was especially pronounced in the case of the SAM10 sample, which exhibited a Pr value of 68 mC·m^−2^ at 100 kHz, whereas the Au sample without a SAM or a PEDOT-PEG lauryl layer exhibited a very small Pr value of 2.4 mC·m^−2^.

The SAM12 sample exhibited relatively low Pr values in the switching frequency range of 10 Hz–10 kHz. A comparison of the surface morphologies in Figure 1 reveals that the SAM12 sample exhibits an obviously different morphology of needle-like crystallites, whereas the other samples exhibit grain-like crystallites. These morphological differences may be related to the lower Pr values of the SAM12 sample. Furthermore, the SAM10 samples exhibited larger grain-like crystallites, which may be related to the larger Pr values at higher switching frequencies. In a previous study by Park *et al.*, a SAM interlayer between the Au electrode and P(VDF-TrFE) led to a structural change related to the Pr performance [7].

**Figure 3:**
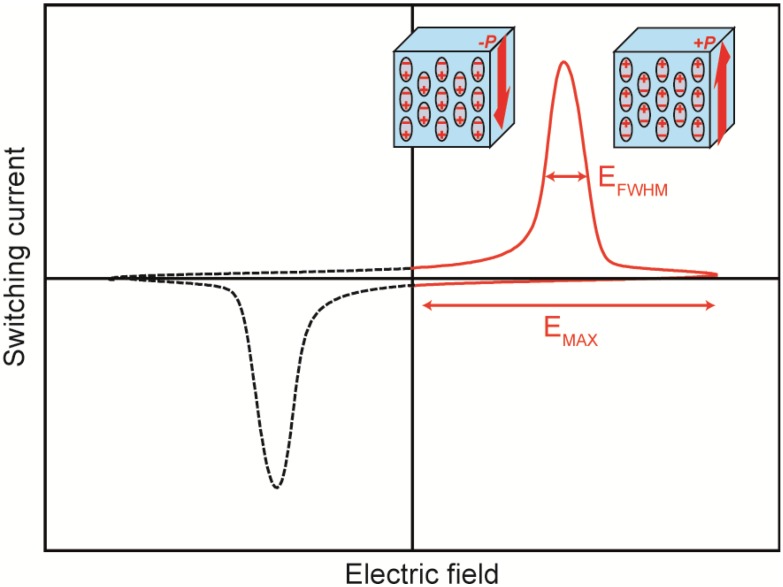
Schematic of the *J*(*t*)-*E* plots used to evaluate the switching time.

As discussed in our previous report [5], the switching speed is an important characteristic for evaluating memory devices. We used the same procedure reported in our previous report [5] to evaluate the switching characteristics of the samples. We evaluated the switching speed using the full-width at half-maximum (FWHM) of the switching current in current (*J*(*t*)) *vs.* applied electric field (*E*) plots. All switching speeds were measured in saturated electric fields of 110–120 MV·m^−1^. The switching speed was evaluated from the inverse of the switching time. The switching time (*t_s_*) was evaluated from Equation (2):

(2) $$t_{S}=\frac{E_{\text{FWHM}}}{E_{\text{MAX}}}\times \frac{t}{4}$$

where *E*_FWHM_ is the full-width at half-maximum of the switching current, *E*_MAX_ is the maximum amplitude of the switching field, and *t* is the time required for one switching cycle. Figure 3 shows a schematic of the *J*(*t*)-*E* plots used to evaluate the switching time.

**Figure 4:**
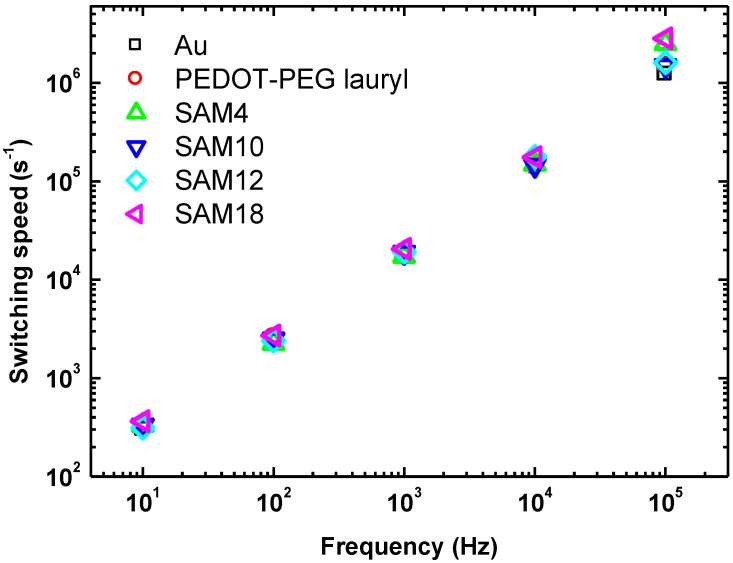
Plot of switching speed as a function of frequency for each sample.

The switching speed is plotted as a function of the cycling frequency for each sample in Figure 4. At lower frequencies, the difference among the Au, PEDOT-PEG, and SAM samples was small; however, at higher frequencies (greater than 10 kHz), significant differences were measured. At 100 kHz, switching speeds of 2.5 × 10^6^ s^−1^ for SAM4 and 2.8 × 10^6^ s^−1^ for SAM18 were measured; these switching speeds are twice as fast as those of the other samples, which had values of 1.2–1.5 × 10^6^ s^−1^. These switching speeds were measured under applied fields of 110 and 120 MV·m^−1^, which were approximately 2.5 times weaker than the previously applied field of 278 MV·m^−1^ [5]. The coercive field usually determines the switching speed. A SAM interlayer leads to a decrease in the coercive field, irrespective of the unchanged switching speed, in the frequency range of 10–10^4^ Hz. The results of leakage-current measurements are discussed in the next paragraph.

**Figure 5:**
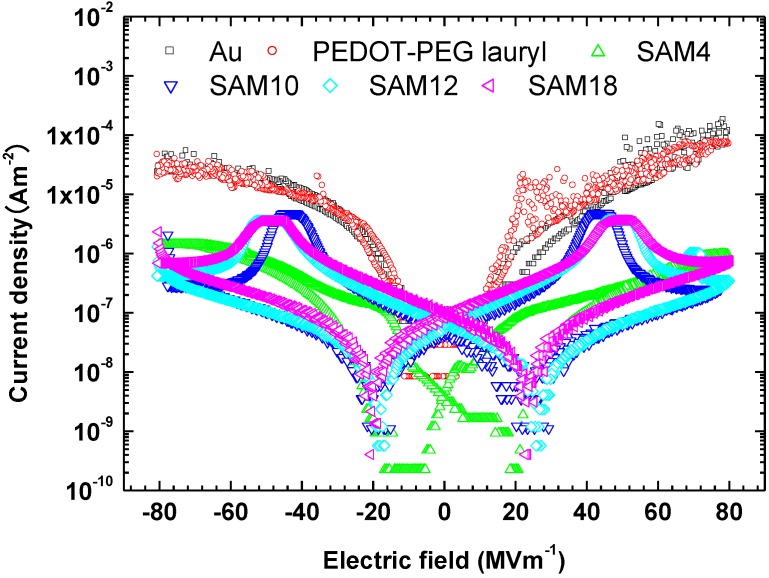
J–E plots showing the leakage currents measured from −80 to 80 MV·m^−1^ for each sample.

**Figure 6:**
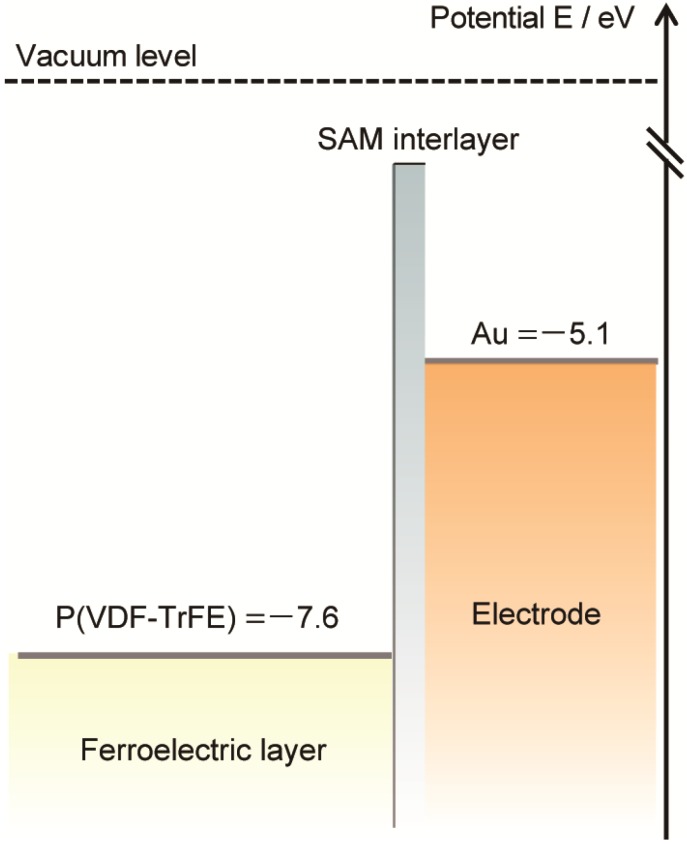
Energy-level diagram of the Au electrode, SAM-Au, and P(VDF-TrFE).

Interlayers between gold electrodes and P(VDF-TrFE), such as the organic conductor PEDOT-PEG lauryl and self-assembled monolayers, significantly change the ferroelectric switching characteristics of P(VDF-TrFE). The question arises as to why the interlayers change those of P(VDF-TrFE). The leakage currents measured for all samples are shown in Figure 5. The leakage current for the sample with the PEDOT-PEG lauryl interlayer is almost the same as that for the sample with an unmodified gold electrode, whereas the leakage currents for the samples with SAM layers are two orders of magnitude lower than those for the unmodified gold electrode and the electrode with a PEDOT-PEG lauryl interlayer. The large decrease in the leakage current is attributed to the creation of an effective energy barrier for charge carriers via the insertion of a SAM interlayer between the Au electrode and the organic P(VDF-TrFE) film. Boer *et al.* reported that SAMs of hexadecanethiol decreased the work functions of silver and gold electrodes by 0.6 eV and 0.1 eV, respectively, resulting in a potential barrier for hole injection into MEH-PPV organic electronic devices [8]. Kinashi *et al.* decreased the large dark current of a photorefractive polymer composite using a SAM-ITO electrode [9]. Figure 6 illustrates the energy-level diagrams of the present P(VDF-TrFE) organic ferroelectric, the Au electrode, and the SAM-Au electrode.

**Figure 7:**
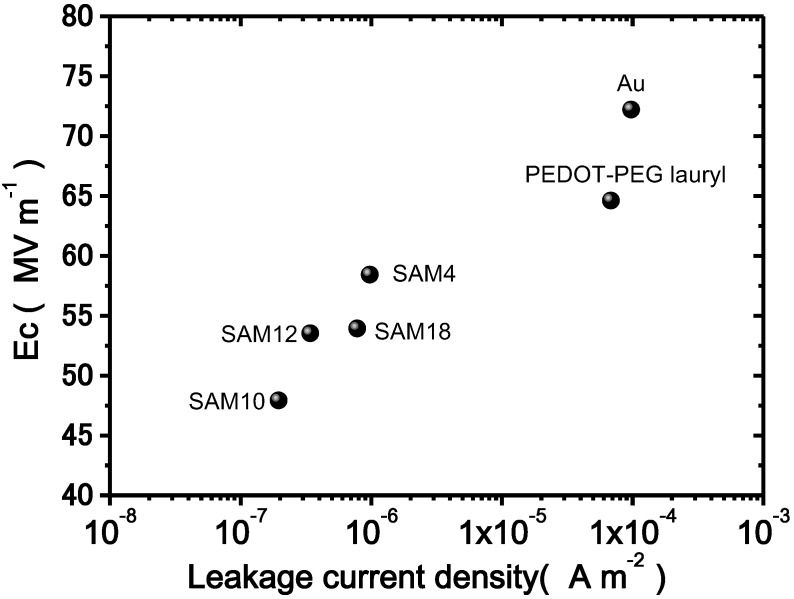
Plots of the leakage current density *vs.* Ec measured under an applied field of 80 MV·m^−1^ and at a cycling frequency of 10 Hz.

Figure 7 shows a plot of the Ec as a function of the leakage current density under an electric field of 80 MV·m^−1^ and a cycling frequency of 10 Hz. As the leakage current density decreases, Ec decreases. For other cycling frequencies, similar relationships were observed. Thus, the leakage current is related to the Ec value. The results clearly demonstrate that the SAM interlayers depress the leakage current density and the Ec values. One possible explanation for the lower coercive field due to insertion of a SAM interlayer is the depression of the effective electric field loss. The SAM interlayers, as previously discussed, greatly decrease the leakage current density. Specifically, the decreased leakage current density leads to the preservation of the effective electric field at the interface between the electrode and the bulk, whereas a larger leakage current does not. Thus, a lower coercive field is measured because of the preserved effective electric field in the samples with SAM interlayers.

## 4. Conclusions

Significant changes in the ferroelectric switching characteristics of the ferroelectric copolymer P(VDF-TrFE) were achieved via the introduction of SAMs between gold electrodes and the copolymer. The organic conductor PEDOT-PEG lauryl also changed the ferroelectric performance. In particular, sample SAM10 exhibited an extremely high-speed switching performance at 100 kHz. Pr values of 68 mC·m^−2^ were achieved at a cycling frequency of 100 kHz. These high performances were not achieved in the absence of a SAM. The switching speeds of the SAM-Au electrodes were twice as fast as those of the unmodified electrode. The large potential barriers formed by the introduction of SAMs between the Au electrodes and the ferroelectric effectively changed the ferroelectric switching characteristics.
